# Notoginsenoside R1 Inhibits Porcine Deltacoronavirus Infection In Vitro by Restoring SERCA2-Mediated Calcium Homeostasis

**DOI:** 10.3390/ani16121836

**Published:** 2026-06-14

**Authors:** Jialu Zhang, Yuqian Liu, Wenzhe Liu, Zhouyuan Wang, Hanlu Wang, Xuejing Xia, Lianci Peng, Tingting Chen, Rendong Fang

**Affiliations:** 1 Joint International Research Laboratory of Animal Health and Animal Food Safety, College of Veterinary Medicine, Southwest University, Chongqing 400715, China; zjl20240745@swu.edu.cn (J.Z.); penglianci@swu.edu.cn (L.P.); 2 Kunming Hemeihua Feed Limited Company, Kunming 682100, China; 3 Department of Molecular Pharmacology, Groningen Research Institute of Pharmacy, University of Groningen, 9713AV Groningen, The Netherlands

**Keywords:** Notoginsenoside R1, porcine deltacoronavirus, cellular calcium ions, endoplasmic reticulum

## Abstract

Porcine coronavirus is one of the prevalent enteric coronaviruses in pigs, causing watery diarrhea and even death in suckling piglets and resulting in giant losses to the pig industry. However, effective antiviral strategies against PDCoV remain limited. Notoginsenoside R1 (NG-R1), a saponin extracted from *Panax notoginseng*, exhibits diverse bioactivities, but its antiviral potential has not been fully characterized. Herein, we systematically investigated the anti-PDCoV effect of NG-R1 and its underlying mechanism. NG-R1 showed no cytotoxic effect on LLC-PK1 cells and exerted antiviral ability against PDCoV infection through targeting the whole life cycle of the virus. In addition, network pharmacology analysis identified calcium signaling as a potentially relevant pathway involved in the antiviral activity of NG-R1. Further data demonstrated that PDCoV infection disrupted intracellular calcium homeostasis, whereas NG-R1 treatment partially restored calcium balance and attenuated endoplasmic reticulum (ER) stress. Moreover, NG-R1 modulated the expression of SERCA2, a key regulator of ER calcium transport. Thapsigargin, an inhibitor of SERCA2, showed similar antiviral capacity to NG-R1. Collectively, our findings suggest that NG-R1 exerts antiviral activity against PDCoV, potentially through regulation of calcium homeostasis mediated by SERCA2. This study provides a theoretical basis for the development of novel antiviral agents targeting calcium signaling pathways.

## Simple Summary

Porcine deltacoronavirus (PDCoV) is a globally prevalent enteric virus that causes severe watery diarrhea and mortality in suckling piglets, threatening the development of the pig industry and public health. Because of the characteristics of its genome, effective vaccines and antiviral therapies against PDCoV remain unavailable. In this study, we evaluated the anti-PDCoV effect of Notoginsenoside R1 (NG-R1), a major bioactive saponin derived from the traditional Chinese herb *Panax notoginseng*. By conducting network pharmacology analysis and calcium imaging, we demonstrated the underlying antiviral mechanism of NG-R1, in which NG-R1 alleviated PDCoV-induced calcium dysregulation in endoplasmic reticulum (ER) by targeting the pump protein SERCA2. These findings highlight the antiviral potential of NG-R1 and provide new insights into therapeutic strategies for PDCoV control.

## 1. Introduction

Coronaviruses belong to enveloped viruses with single-stranded positive-sense RNA, and are characterized by a wide range of hosts and acute potential risks to humans and animals [1]. Swine enteric coronaviruses (SeCoVs) are major coronaviruses causing porcine diarrhea and other related symptoms, composed of porcine epidemic diarrhea virus (PEDV), porcine deltacoronavirus (PDCoV), transmissible gastroenteritis virus (TGEV), and swine acute diarrhea syndrome coronavirus (SADS-CoV). PDCoV has been broadly reported in the world since its outbreak in the US in 2014, whereas a retrospective study revealed that the earliest PDCoV infection could be traced back to 2009 [2]. Due to the weaker mortality in pigs than PEDV, the epidemiological impact and associated risks of PDCoV were initially underestimated. Nowadays, PDCoV infection has developed into a global problem for the pig industry and public health, which was hinted by its detection in several Haitian children with fever [3]. Hence, the investigation of an anti-PDCoV candidate drug is urgent for PDCoV control. Nowadays, natural products have been shown as a potential library for antiviral candidate drugs; for example, 25-hydroxycholesterol and resveratrol were demonstrated to have anti-PDCoV abilities [2].

During the infection, manipulation of the endoplasmic reticulum (ER) by pathogens always induces ER stress [4]. To address the abnormal ER stress, cells activate unfolded protein response (UPR), including PKR-like ER kinase (PERK) signaling, transcription factor 6 (ATF6) signaling, and inositol-requiring enzyme 1 (IRE1) signaling, to restore the ER balance [5]. Various viral infections were verified to induce ER stress by detecting the UPR activation, such as SARS-CoV-2, African swine fever virus, and porcine reproductive and respiratory syndrome virus (PRRSV) [6,7,8]. A recent study also identified the UPR activation in PDCoV infection in LLC-PK1 cells; furthermore, the ATF6 signaling benefited the PDCoV replication [9]. Ca^2+^ was involved in various cellular processes, and ER is the most important storage organelle in the cell [10]. During viral infection, calcium signaling was utilized by the envelope protein to facilitate the SARS-CoV-2 replication [11]. However, the deep mechanism by which ER stress affected the PDCoV infection and the role of calcium signaling in PDCoV infection were still unknown.

Notoginsenoside-R1 (NG-R1) is one of the major bioactive saponins isolated from *Panax notoginseng* (PN), which is a medicinal herb and mainly distributed in Yunnan province, China [12]. Previous studies have identified multiple biological activities of PN, including anti-inflammatory and antidepressant effects [13] and ameliorating ulcerative colitis [14]. In recent years, diverse pharmacological effects of NG-R1 have been discovered, including bone remodeling [15], cardiovascular protection [16], and neuroprotective effects [17]. Several studies indicated that NG-R1 might target ER stress to alleviate cell apoptosis [18,19]. In addition, several studies indicated the anti-infection abilities of NG-R1, for example, HBV infection in HepG2 and MHCC97H cells was inhibited by NG-R1 treatment, and the downregulated activity of SIRT1 was involved in the anti-HBV mechanism [20]. Moreover, in an in vivo sepsis model constructed by cecum ligation puncture and an in vitro sepsis model induced by lipopolysaccharide stimulation, NG-R1 repaired the intestinal barrier and promoted the prognosis by inhibiting Drp1-mediated mitochondrial fragmentation [21]. However, the antiviral effect of NG-R1 on PDCoV and the underlying mechanism are still yet to be elucidated.

In this study, we aimed to investigate the antiviral potential of NG-R1 against Porcine deltacoronavirus infection and to elucidate its underlying molecular mechanisms. Given the critical role of calcium homeostasis in viral replication and host cellular stress responses, we focused on the involvement of endoplasmic reticulum (ER)-associated calcium regulation. In particular, we explored whether NG-R1 modulates SERCA2-mediated ER calcium dynamics and its potential impact on PDCoV infection. This study provides new insights into the interplay between viral infection, calcium signaling, and ER stress, and may offer a theoretical basis for developing novel antiviral strategies targeting host calcium homeostasis.

## 2. Materials and Methods

### 2.1. Cell Culture and Virus

LLC-PK1 cells (ATCC CL-101) were cultured in Dulbecco’s modified Eagle’s medium (DMEM) (Gibco, Gaithersburg, MD, USA) supplemented with 1% antibiotic-antimycotic (Gibco, Gaithersburg, MD, USA), 1% nonessential amino acids solution (NEAA) (Gibco, Gaithersburg, MD, USA), and 10% fetal bovine serum (FBS) (Gibco, Fitzroy North, Victoria, Australia). The cells were cultured and maintained at 37 °C with 5% CO_2_. The PDCoV CHN-HN-1601 strain (GenBank No. MG832584) was generously offered by Hanchun Yang, China Agricultural University, Beijing, China, and maintained in our lab.

### 2.2. Reagents

The TransStart one-step gDNA removal and cDNA synthesis supermix kit (AT311-02) and TransStart Tip Green qPCR supermix (AQ142-21) were ordered from TransGen Biotech (Beijing, China). The RNAiso Easy (TCH020) was ordered from Takara (Dalian, China). Notoginsenoside-R1 (HY-N0615), and Thapsigargin (HY-13433) were purchased from MedChemExpress (Monmouth Junction, NJ, USA). DAPI (49,6-diamidino-2-phenylindole) (C0060) was ordered from Solarbio Science & Technology (Beijing, China). Fluo-4 calcium detection kit (S1061S), and ER-Tracker Red (C1041S) were purchased from Beyotime (Shanghai, China).

### 2.3. Antibodies

ATF6 (24169-1-AP), ATF4 (10835-1-AP), GAPDH (60004-1-Ig), HRP-conjugated Goat Anti-Mouse IgG(H + L) (SA00001-1) and HRP-conjugated Affinipure Goat Anti-Rabbit IgG(H + L) (SA00001-2) were purchased from Proteintech Group (Rosemont, IL, USA). IP3R (ET1704-77), SERCA2 (ET1703-01) and GRP78 (ER1706-50) were ordered from HUABIO (Hangzhou, Zhejiang Province, China). AF488-labeled goat anti-mouse IgG (H + L) (A0428) was purchased from Beyotime (Shanghai, China). PDCoV N (PDCoV11-F) was obtained from Alpha Diagnostic International (San Antonio, TX, USA).

### 2.4. Viral Titers Detection

The median tissue culture infectious dose (TCID_50_) assay was performed to determine viral titers. Briefly, cells were seeded into 96-well plates and cultured overnight until reaching full confluence. The supernatants were serially diluted 10-fold using DMEM medium; after that, the diluted supernatants were inoculated into cell monolayers, with 8 replicate wells set for each dilution to minimize experimental error. After continued incubation for several days, the morphological changes of cells in each well were examined individually using an inverted microscope (Nikon, Tokyo, Japan) to observe cytopathic effects. The number of wells showing cytopathic effects was recorded and utilized to calculate viral titers using the Reed–Muench method.

### 2.5. RNA Extraction and Quantitative Real-Time PCR

According to the manufacturer’s protocols, total RNA from cells was isolated using RNAiso Easy, and then cDNA was reverse-transcribed using a cDNA synthesis kit. The amplification was performed using qPCR supermix in triplicate on the CFX-96 (Bio-Rad, Hercules, CA, USA). The relative analysis results were normalized to the expression of housekeeping gene GAPDH and calculated using the comparative CT method (2^−ΔΔCT^). Primers designed specifically for genes are listed in Table 1.

### 2.6. Western Blot Analysis

Harvested cells were placed on ice and lysed using RIPA buffer mixed with 1% protease inhibitor cocktail to prevent protein degradation. The protein supernatants were collected into fresh tubes. Subsequently, the extracted proteins were separated by sodium dodecyl sulfate-polyacrylamide gel electrophoresis (SDS-PAGE) and electrophoretically transferred onto 0.45 μm polyvinylidene fluoride (PVDF) membranes (Millipore, Billerica, MA, USA). To block non-specific binding sites, the membranes were incubated in 5% skim milk (A600669, Sangon Biotech, Shanghai, China) at room temperature for 2 h. After that, the membranes were probed with the specified primary antibodies and incubated overnight at 4 °C. The next day, the membranes were washed and incubated with horseradish peroxidase (HRP)-conjugated secondary antibodies for 1 h at room temperature. Finally, protein bands were visualized using an enhanced chemiluminescence (ECL) detection system (Tanon 6200 imaging workstation, Tanon Science & Technology, Shanghai, China), and the relative intensity of the bands was quantified using Image J software (version 1.52a).

### 2.7. Cell Viability Assay

The cell viability was measured using the cell counting kit-8 (C0038, Beyotime, Shanghai, China) in accordance with the manufacturer’s protocol. Briefly, cells were seeded into 96-well plates at an appropriate density and incubated overnight at 37 °C with 5% CO_2_. After treatment by chemicals, 10 μL CCK-8 reagent was added to each well, and the incubation lasted for 1–4 h. A microplate reader was used to measure the absorbance of each well at a wavelength of 450 nm.

### 2.8. Immunofluorescence Assay

Cells subjected to diverse experimental treatments were fixed in 4% paraformaldehyde at room temperature for 30 min, followed by three rounds of washing with phosphate-buffered saline (PBS) to remove residual fixative. For membrane permeabilization, the fixed cells were incubated with 0.1% Triton X-100 at room temperature for 15 min. Non-specific binding sites were blocked by incubating the cells in PBS supplemented with 5% bovine serum albumin (BSA) (9048-46-8, Solarbio, Beijing, China) at 37 °C for 1 h. The cells were then probed with the relevant primary antibodies, incubated overnight at 4 °C, and subsequently incubated with AF488-conjugated secondary antibodies at room temperature for another 1 h to enable fluorescence labeling. After three additional washes with PBS to eliminate unbound antibodies, cell nuclei were counterstained with DAPI. Fluorescence images were captured using a Nikon fluorescence microscope (Nikon, Tokyo, Japan).

### 2.9. Network Pharmacology Prediction

A total of 917 potential targets of NG-R1 were collected from the ITCM database. Meanwhile, 7332 viral diarrhea-related targets were retrieved from the GeneCards database (https://www.genecards.org/ (accessed on 20 January 2026)) using the keyword “viral diarrhea”. The intersecting genes were analyzed via an online bioinformatics platform, yielding 237 overlapping targets. These 237 common targets were submitted to the DAVID database for Gene Ontology (GO) and Kyoto Encyclopedia of Genes and Genomes (KEGG) enrichment analyses, identifying key biological processes (BP), cellular components (CC), and critical signaling pathways underlying the antiviral activity of NG-R1. The top GO terms and KEGG pathways were visualized as bar plots and bubble plots, respectively. In addition, the network among 237 overlapping targets and signaling pathways was visualized using Cytoscape 3.9.1.

### 2.10. Treatment of Cells with Inhibitors

LLC-PK1 cells were inoculated with PDCoV (MOI = 0.1) for 1 h at 37 °C, during which the different inhibitors were added simultaneously. After the unabsorbed viruses were removed, the cells were further cultured in medium containing the respective inhibitors, an equivalent concentration of solvent (dimethylsulfoxide, DMSO), or plain DMEM. At 24 hpi, the cells were harvested and prepared for subsequent qRT-PCR and Western blot.

### 2.11. Staining of Calcium Ions and Endoplasmic Reticulum

After pretreatment of cells with PDCoV infection, every well in the plate was washed by PBS. Fluo-4 AM was diluted in a related solvent and incubated with cells at 37 °C for 30 min in the dark to stain cellular ionic calcium. After staining, cells were washed twice with PBS to eliminate unloaded probe. Fluorescence images were immediately captured using a Nikon microscope (Nikon, Tokyo, Japan).

Similar to the staining of calcium ions, the pretreated cells were washed twice by PBS before the staining. Then, ER-Tracker Red was diluted in medium and incubated with cells at 37 °C for 20 min in the dark. After that, the cells were washed twice with PBS to remove excess stain. ER-stained images were obtained using a fluorescence microscope (Nikon, Tokyo, Japan).

### 2.12. Statistical Analysis

Statistical differences were determined by one-way ANOVAs or Student’s *t*-test performed using GraphPad Prism 9.0 software (GraphPad Software 9.0, La Jolla, CA, USA). For all experiments, differences were considered to be statistically significant when the corresponding *p*-values were <0.05.

## 3. Results

### 3.1. NG-R1 Significantly Inhibits PDCoV Replication

**Figure 1:**
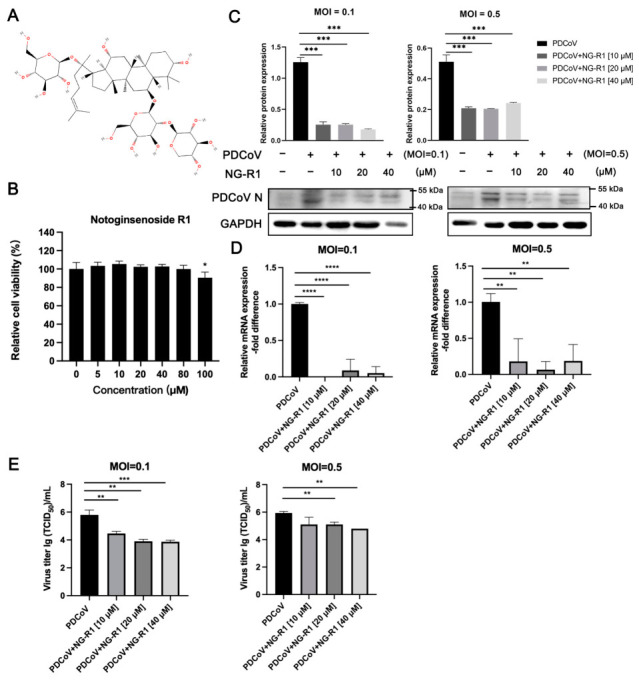
NG-R1 significantly inhibits PDCoV replication. (**A**) The 2D chemical structure of NG-R1. (**B**) LLC-PK1 cells were treated with NG-R1 at concentrations from 0 to 100 μM for 24 h. The relative cell viability was detected by using CCK-8 assay. (**C**–**E**) The LLC-PK1 cells were infected by PDCoV at different MOIs of 0.1 and 0.5, followed by addition of NG-R1 at the concentrations of 10, 20 and 40 μM. (**C**) Samples were collected at 24 hpi, and PDCoV replication, indicated by level of PDCoV N, was detected by Western blotting. The immunoblotting bands were calculated by Image J. (**D**) The transcript level of *PDCoV N* was measured by qRT-PCR. (**E**) The virus titers of the cell supernatant were evaluated by TCID50 analysis. Data were shown as means ± SD from three independent experiments. * *p* < 0.05; ** *p* < 0.01; *** *p* < 0.001; **** *p* < 0.0001.

The 2D chemical structure of NG-R1 was shown in Figure 1A. To investigate the antiviral effect of NG-R1, we first evaluated the cytotoxic effect of NG-R1 on LLC-PK1 cells. The cells were incubated with NG-R1 at concentrations from 5 to 100 μM for 24 h, and the relative cell viability was detected by using the cell counting kit-8 (CCK-8) assay (Figure 1B). The treatment of NG-R1 at a concentration of less than 80 μM showed no cytotoxic effects. Thus, nontoxic concentrations of NG-R1 (10, 20 and 40 μM) were chosen to determine the antiviral abilities. The protein expression levels of PDCoV N were detected by Western blot, which showed that NG-R1 significantly inhibited the expression of PDCoV N after infection with different MOI in a dose-independent manner (Figure 1C). In addition, the qRT-PCR analysis verified that the treatment of NG-R1 is effective for the inhibition of PDCoV N transcription (Figure 1D). Moreover, the viral titration assay further indicated the antiviral effect of NG-R1 against the progeny virus production of PDCoV (Figure 1E). These data demonstrated the antiviral capacity of NG-R1 against PDCoV replication.

### 3.2. NG-R1 Suppresses the Whole Life Cycle of PDCoV

**Figure 2:**
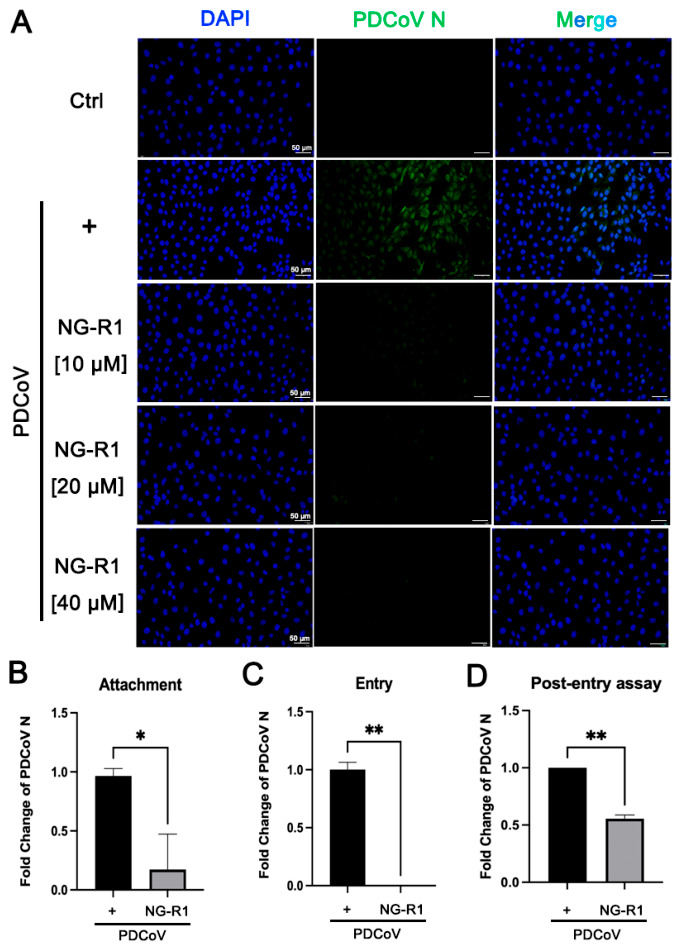
NG-R1 suppresses the whole life cycle of PDCoV. (**A**) The inhibitory effect of NG-R1 on PDCoV was further assessed by immunofluorescence staining. PDCoV N protein was stained green and the cellular nuclei were stained blue. Scale bar, 50 μm. (**B**,**C**) The NG-R1 was added into LLC-PK1 cells during the PDCoV infection stages, including attachment (**B**), entry (**C**), and post-entry (**D**). The transcript of *PDCoV N* was measured by qRT-PCR. Data were expressed as means ± SD from three independent experiments. * *p* < 0.05; ** *p* < 0.01.

The indirect fluorescence staining of PDCoV N was applied to visually evaluate the PDCoV infection after the treatment of NG-R1. Similar to the data in Figure 1, we found that the treatment of NG-R1 significantly reduces the fluorescent intensity of viral protein, and the application of 40 μM NG-R1 showed the best inhibitory effect (Figure 2A). To further confirm the concrete stage of PDCoV targeted by NG-R1, the virus incubation conditions with LLC-PK1 cells at different temperatures and times were divided into viral attachment (4 °C for 1 h), viral entry (37 °C for 1 h after viral attachment), and viral post-entry (37 °C for 12 h after viral entry). The qRT-PCR analysis of *PDCoV N* was applied to imply the replication level of PDCoV. As shown in Figure 2B–D, the addition of NG-R1 at different stages exhibited consistently inhibitory effects on the PDCoV life cycle. These data indicated the great antiviral potential of NG-R1.

### 3.3. Network Pharmacology Analysis Indicates Role of Calcium Signaling Pathway in the Antiviral Activity of NG-R1

**Figure 3:**
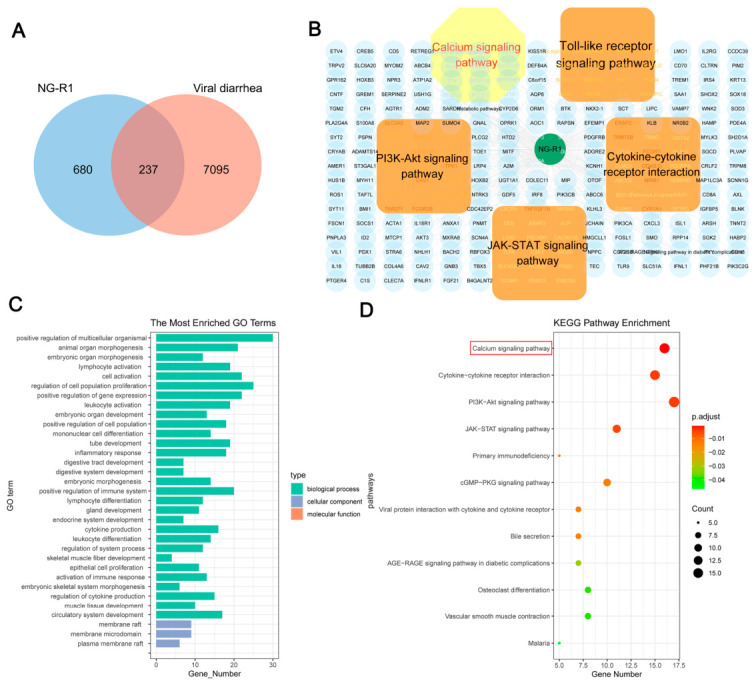
Network pharmacology analysis indicates role of calcium signaling pathway in the antiviral activity of NG-R1. (**A**) NG-R1- and viral diarrhea-related genes were collected from indicated databases, and a Venn diagram was created to reflect intersecting genes. (**B**) The network between signaling pathways and targets was constructed by Cytoscape 3.9.1. (**C**,**D**) GO analysis and KEGG enrichment were displayed.

In order to get the general prediction results, we retrieved the Genecards database with viral diarrhea and acquired 7332 related genes. The targets of NG-R1 were obtained from the ITCM database. The intersection analysis indicated that 237 genes were involved in the therapeutic effect of NG-R1 on viral diarrhea (Figure 3A). After that, the GO and KEGG enrichment were performed according to these intersecting genes, and a network involving the NG-R1, potential targets, and related pathways was constructed using Cytoscape 3.9.1 (Figure 3B). The GO enrichment revealed that NG-R1 mainly targeted the genes related to biological processes and cellular components. In detail, the antiviral ability of NG-R1 might be related to processes including positive regulation of multicellular organismal processes, animal organ morphogenesis, lymphocyte activation, inflammatory response, positive regulation of the immune system, cytokine production, membrane microdomain, and membrane raft (Figure 3C). The cytokine–cytokine receptor interaction, PI3K-Akt signaling pathway, and JAK-STAT signaling pathway were enriched in KEGG enrichment; moreover, the calcium signaling pathway was among the most enriched pathways, which implied its importance (Figure 3D).

### 3.4. PDCoV Infection Stimulates Calcium Ion Accumulation in Endoplasmic Reticulum

**Figure 4:**
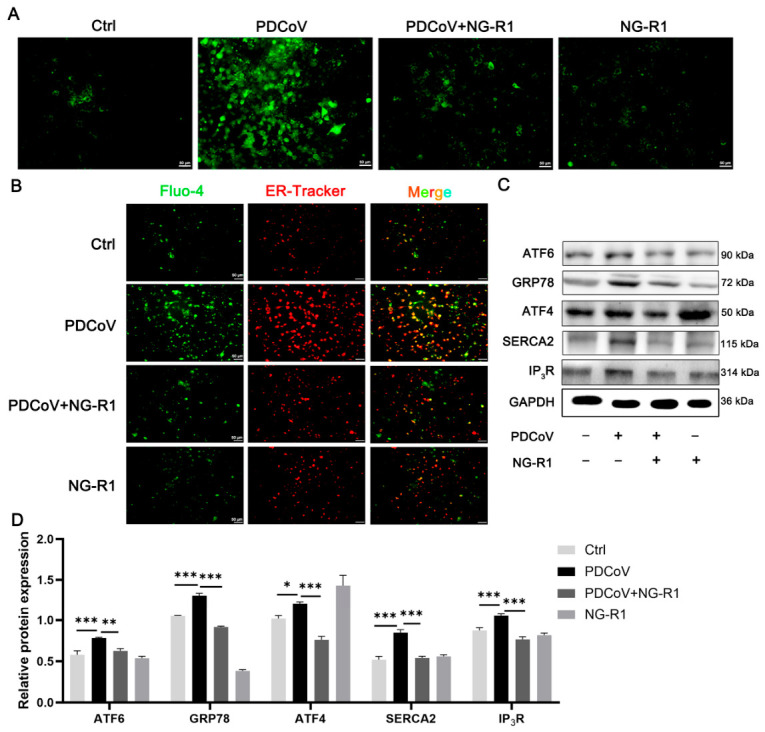
PDCoV infection stimulates calcium ion accumulation in the endoplasmic reticulum. (**A**–**D**) LLC-PK1 cells were infected with PDCoV (MOI = 0.1) and treated with 40 μM NG-R1. The cells were processed at 24 hpi. (**A**) Calcium ions were stained green with Fluo-4 AM. Scale bar, 50 μm. (**B**) The cells were stained with Fluo-4 AM and ER-Tracker at the same time. The calcium ions were stained green and ERs were stained red. Scale bar, 50 μM. (**C**) The expression levels of the indicated proteins were detected by Western blotting. (**D**) The immunoblotting bands in panel C were measured by Image J. Data were expressed as means ± SD from three independent experiments. * *p* < 0.05; ** *p* < 0.01; *** *p* < 0.001.

According to the results obtained from the network pharmacology analysis, we attempted to investigate the role of the calcium pathway in PDCoV infection and the therapeutic effect of NG-R1. The Fluo-4 AM staining kit was applied to detect the calcium ion levels after PDCoV infection and NG-R1 treatment. The results showed that PDCoV infection induces the accumulation of calcium ions, and treatment with NG-R1 significantly decreased the calcium ion levels at 24 hpi (Figure 4A). A previous study had pointed out that the endoplasmic reticulum (ER) is the most important place to maintain the balance of cellular calcium ions. To detect the calcium ion levels in the ER after infection with PDCoV, we applied a Fluo-4 AM staining kit and ER-Tracker simultaneously to colocalize calcium ions and ER. The staining result suggested that PDCoV infection induces abundant colocalization of calcium ions and ER, which was shown in yellow at the Merge channel. The treatment of NG-R1 after PDCoV infection obviously reduced the amount of colocalization (Figure 4B). Then, we detected the protein expression levels of ATF6, GRP78 and ATF4, which were related to the ER stress, and the levels of SERCA2 and IP3R, which were related to the balance of ER calcium ions (Figure 4C). The expression tendency of ATF6, GRP78 and ATF4 was consistent with SERCA2 and IP3R, which were upregulated in PDCoV-infected cells compared with the control group and were decreased after the NG-R1 treatment (Figure 4D).

### 3.5. Inhibition of SERCA2 Contributes to the Resistance of PDCoV by NG-R1

**Figure 5:**
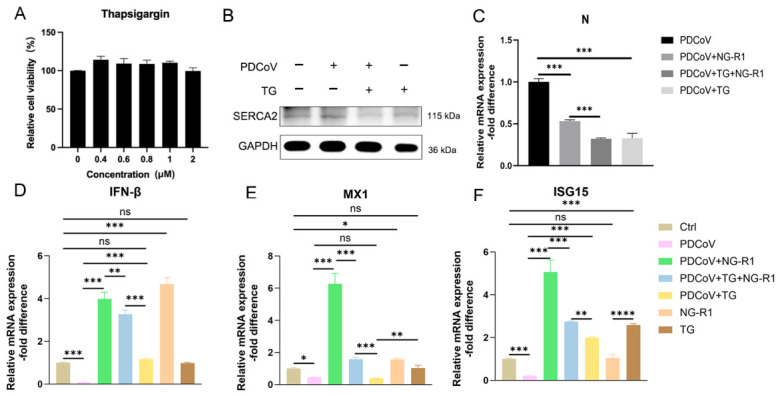
Inhibition of SERCA2 contributes to the resistance of PDCoV by NG-R1. (**A**) LLC-PK1 cells were treated with Thapsigargin (TG) of different concentrations for 24 h, and the relative cell viability was measured by CCK-8 kit. (**B**) LLC-PK1 cells were infected with PDCoV (MOI = 0.1) and treated with 1 μM TG. The protein expression level of SERCA2 was detected by Western blotting. (**C**) LLC-PK1 cells were infected with PDCoV (MOI = 0.1); the TG and NG-R1 were added individually or simultaneously. At 24 hpi, the cells were harvested for qRT-PCR analysis. (**D**–**F**) The cells were treated the same as channel C. The mRNA expressions of *IFN-β*, *MX1* and *ISG15* were analyzed by qRT-PCR. Data were expressed as means ± SD from three independent experiments. * *p* < 0.05; ** *p* < 0.01; *** *p* < 0.001; **** *p* < 0.0001; ns not significant.

To investigate the role of SERCA2 in PDCoV infection, we applied Thapsigargin (TG), broadly used as an inhibitor of SERCA2, to detect the expression of PDCoV N and the change in IFN-related genes. The cytotoxic effect of TG on LLC-PK1 cells was measured by CCK-8 analysis, and TG showed no toxic effect at the concentration range of 0.4–2 μM (Figure 5A). In the following experiment, 1 μM TG was applied. The inhibitory ability of TG to SERCA2 was confirmed by Western blotting, and the application of TG during PDCoV infection also suppressed the expression of SERCA2 (Figure 5B). We found that TG inhibited PDCoV replication individually and showed great antiviral ability when administered with NG-R1 simultaneously (Figure 5C). In addition, the application of TG also raised the expressions of *IFN-β* and *ISG15*, except *MX1*, in the PDCoV infection, and the NG-R1 treatment dramatically increased the expressions of *IFN-β*, *MX1* and *ISG15* after PDCoV infection. The individual addition of NG-R1 significantly raised the expression of *IFN-β* and *MX1*, and the individual addition of TG upregulated the expression of *ISG15* significantly (Figure 5D–F).

## 4. Discussion

Traditional Chinese medicine (TCM) is a great candidate drug pool for the treatment of various diseases, including cancer, hepatopathy, nephropathy and infectious diseases [22,23]. In recent years, different active components of TCM were recognized to have multiple therapeutic effects; however, the pharmacological action of the same medicine in different diseases is still unknown, and the underlying mechanism is still yet to be well demonstrated [24]. Our work firstly identified the antiviral effect of NG-R1 on PDCoV infection and interpreted the possible underlying mechanism. NG-R1 showed an antiviral effect by targeting the whole life cycle of PDCoV. In addition, the abnormal calcium ion level was restored by NG-R1 treatment, and the SERCA2-mediated ER calcium ion accumulation was involved in the antiviral ability of NG-R1.

*Panax ginseng*, a famous medicine in China, has been investigated for a long time for its therapeutic effects in different diseases. PN is similar to *Panax ginseng*, and both of them were classified in the same category. Thus, the common bioactive materials from *Panax ginseng* and PN were discovered to have the same pharmacological activity, such as ginsenosides [25]. The previous studies indicated that ginsenosides exert broad antiviral effects on Human Rhinovirus (HRV) [26], influenza virus [27], and hepatitis viruses [28]. In addition, ginsenosides also showed therapeutic effects on the enteric virus infection, such as Enterovirus 71 [29] and Norovirus [30]. For the control of SARS-CoV-2, ginsenosides also exhibited great antiviral potential [31]. With regard to PN, the research regarding its antiviral capacity mostly concentrates on saponins. In recent years, researchers identified the antiviral effect of PN saponins (PNS) on PEDV infection. PNS suppressed PEDV infection in Vero cells in a dose-dependent manner at 24–48 hpi and mainly targeted the viral replication stage. In addition, PNS treatment in PEDV-challenged cells upregulated multiple gene expressions, such as IFIT1, IFIT3, CFH, IGSF10, ID2, and PLCB4 [32]. However, the antiviral efficacy of PN, except for ginsenosides, was rarely explored. In a recent study, the efficacy of active ingredients of TCM was evaluated as an immunologic adjuvant on the resistance of influenza viruses, including PNS. The immunization with the influenza vaccine and PNS improved the hemagglutination inhibition antibody titers to the influenza vaccine and stimulated the lymphocyte proliferation in the spleen but failed to reduce the spleen index. In addition, PNS activated the bone marrow dendritic cells to the influenza vaccine and decreased the secretion of TNF-α, IL-12 and IL-1β to the influenza vaccine [33]. Our work identified the antiviral effect of NG-R1, a unique saponin of PN, indicating the antiviral potential of PN. The network pharmacology was applied to explore underlying mechanisms; we found that the most enriched pathways contain TLR signaling (one of the Pathogen-associated molecular patterns signaling pathways, PAMP), JAK-STAT signaling (the well-known antiviral signaling pathway), PI3K-AKT signaling and cytokine–cytokine receptor interaction. Moreover, the calcium signaling pathway was one of the top enriched pathways, which is closely related to cellular proliferation and molecular function. The results of the network pharmacology also indicated the potential relationship among these pathways, for example, whether the regulation of calcium affects the immune response via JAK-STAT signaling, which provides novel insight and aids in future experimental design.

Ionic calcium participated in the whole intracellular stages of the virus, including entry, replication, release and viral protein expression [34]. The level of Ca^2+^ manipulated by viral infection further promoted viral replication and consistent infection. The viral proteins targeting components of the calcium signaling pathway to utilize Ca^2+^ and facilitate viral replication are termed viroporins. For example, the HA protein of IAV binds to channel Ca_v_1.2 to promote viral entry [35], and the X protein of HBV binds STIM1-ORAI1 complex to alter Ca^2+^ concentration [36]. With regard to coronavirus, envelope proteins of SARS-CoV and MERS-CoV were identified as ion-channel-possessing functions [37,38]. In addition, PEDV ORF3 was identified as a viroporins [39]. Our work identified the abnormal Ca^2+^ level increase along with the PDCoV infection. The ER plays a key role in maintaining calcium homeostasis, which is related to the induction of ER stress. Recently, NSP4 of rotavirus was shown to release ER Ca^2+^ to promote viral infections and also enhance viral pathogenic ability [40]. Our results showed that PDCoV infection induced calcium accumulation and the expression of ER stress-related proteins, while the NG-R1 treatment attenuated these effects. Furthermore, we also found that NG-R1 possessed the ability to modulate the expression of SERCA2, and the application of TG also suppressed PDCoV replication. These findings suggest that the calcium homeostasis targeted by NG-R1 may help to impede viral replication. IP_3_R is known to regulate ER calcium release to the cytoplasm and maintain ER calcium homeostasis with SERCA2. The expression of IP_3_R showed the same tendency as the expression of SERCA2 and ER-stress-related proteins, indicating that the ER calcium change was regulated by ER stress during PDCoV infection. A previous study indicated that ER-associated calcium was released into the cytoplasm via IP_3_R, and the calcium levels in the cytoplasm and ER were raised during PRRSV infection [41]. The functions of SERCA2 and IP_3_R in ER calcium input and release might have a sequential relationship, which needs to be further elucidated in the future. In addition, we also noticed that the individual application of NG-R1 induced obvious upregulated expression of ATF4 and *IFN-β*. The induction of ATF4 is not a virus-specific marker; however, it plays an important role in the integrated stress response. Under different environmental stresses, the activation of ATF4 was shown to induce autophagy and benefit cell survival [42,43]. Moreover, dietary phytochemicals were shown to induce different levels of stress to help cells tolerate environmental stresses [44]. PDCoV is a kind of immune-escape virus, which results in the inhibition of the immune response. Our data indicated that PDCoV inhibited the expression of *IFN-β*, *MX1* and *ISG15*, and the treatment of TG and NG-R1 showed immune-enhancing ability, which contributed to the antiviral capacity of TG and NG-R1. Therefore, combined with the cytotoxic effect results, the upregulated ATF4 and *IFN-β* by NG-R1 may represent a protective response.

Despite these findings, we failed to investigate the direct molecular interaction between NG-R1 and SERCA2; thus, the precise mechanism also needs to be further demonstrated. The antiviral efficacy of NG-R1 also needs an in vivo model to be verified.

## 5. Conclusions

In conclusion, this study showed the anti-PDCoV potential of NG-R1 *in vitro*, the data demonstrated the close relationship between Ca^2+^ homeostasis and virus replication and that SERCA2 might be involved in the antiviral effect of NG-R1 to restore the homeostasis of cellular Ca^2+^. These findings provide new insights into the role of calcium signaling in PDCoV infection and highlight NG-R1 as a potential candidate for the prevention and control of diarrheal diseases in piglets. Further studies are warranted to elucidate the precise molecular mechanisms and to evaluate its therapeutic potential in vivo.

## Figures and Tables

**Table 1:** Primers used for qPCR.

Gene	Direction	Sequence (5′ → 3′)	Production Size
PDCoV N	Forward	AACTTTCAGGCAGGGGCAAT	134 bp
	Reverse	GGTTTGGTGGGTGGCTCATA	
GAPDH	Forward	ACATGGCCTCCAAGGAGTAAGA	106 bp
	Reverse	GATCGAGTTGGGGCTGTGACT	
IFN-β	Forward	TTCGAGGTCCCTGAGGAGATT	176 bp
	Reverse	TCCATCTGCCCATCAAGTTCC	
MX1	Forward	GTGGAGAAAAGTCACAAAACAGGGC	288 bp
	Reverse	TTTGCCCTTCCATTCGTCTTCT	
ISG15	Forward	GGTGAGGAACGACAAGGGTC	177 bp
	Reverse	GGCTTGAGGTCATACTCCCC	

## Data Availability

The raw data supporting the conclusions of this article will be made available by the authors on request.

## Abbreviations

The following abbreviations are used in this manuscript:

PDCoV	Porcine deltacoronavirus
NG-R1	Notoginsenoside R1
ER	Endoplasmic reticulum
SeCoVs	Swine enteric coronaviruses
PEDV	porcine epidemic diarrhea virus
TGEV	transmissible gastroenteritis virus
SADS-CoV	swine acute diarrhea syndrome coronavirus
PN	*Panax notoginseng*
UPR	unfolded protein response
PERK	PKR-like ER kinase
ATF6	transcription factor 6
IRE1	inositol-requiring enzyme 1
PRRSV	porcine reproductive and respiratory syndrome virus
SERCA2	Sarcoplasmic/endoplasmic reticulum Ca^2+^ATPase 2
DMEM	Dulbecco’s modified Eagle’s medium
NEAA	nonessential amino acids solution
FBS	fetal bovine serum
TCID_50_	median tissue culture infectious dose
GO	Gene Ontology
KEGG	Kyoto Encyclopedia of Genes and Genomes
CCK-8	cell counting kit-8
TG	Thapsigargin
TCM	Traditional Chinese medicine
PNS	*Panax notoginseng* saponins
